#  New Trends of Doping in Sport

**Published:** 2010

**Authors:** Farzad Kobarfard

Drugs are designed and developed for medical purposes and they are intended to be beneficial to the human physiological system. However, it is well recognized that drugs could have unwanted side effects which could sometimes be harmful to the patient’s health. Therefore, regulatory authorities all over the world insist on sophisticated and strict scientific and clinical assessment of any drug introduced for medical uses. However, the competitive nature of sport sometimes encourages the athletes to use drugs illicitly with the intention of producing an unfair advantage over their rivals but in these cases, no detailed studies are conducted to assess the benefits and dangers of such application of drugs. It is thus a necessity to control the misuse of drugs in sport, currently referred to as doping. The first action in this regard was taken in 1967 by International Olympic Committees (IOC), by publishing the list of banned substances and methods which comprised five groups: sympathomimetic amines, stimulants of CNS, narcotic analgesics, anti-depressants and major tranquillizers. Anti-depressants and major tranquillizers were removed from the list one year later. In 1976, after the winter Olympic games in Innsbruk, anabolic steroids were added to the list. In 1984 the use of exogenous testosterone was controlled, based on a test that measured the ratio of testosterone and epitestosterone levels in urine. Not only the use of prohibited substances were controlled, but the use of pharmacological, chemical and physical manipulations were also controlled in 1988 after it was revealed that probenecid was effective in reducing the urine concentration of many anabolic steroids. At the same time the use of diuretics and blood transfusions were prohibited. The next major change was in 1989 when the use of a number of hormones was banned including human chorionic gonadotropiroin (HCG), ACTH and human growth hormone (hGH). Erytropoietin (EPO) was added to the list of prohibited substances in 1990. In 1999, the IOC held an international conference on doping and the outcome was the formation of the world Anti-Doping Agency (WADA), an organization which is supported by both sport and governmental authorities. WADA published its first list of prohibited substances in 2004 and it continues to do so on a yearly basis. 

Adding new compounds or new class of compounds to the prohibited list is a rather complex process based on careful monitoring of the reports by WADA-accredited laboratories and consulting with independent research groups all over the world. Regardless of this process, it is clear that the addition of new substances or new class of substances to the prohibited list is the result of advances and successes in the field of drug discovery.

As we are witnessing a shift from classic small molecules towards recombinant drugs in therapeutic arena, the same trend is observed in misuse of drugs in sport. 

Advances in recombinant DNA technology have created one of the most powerful tools in current doping arsenal. Recombinant erythropoietin and hGH are currently being abused, but have been fortunately detectable by employing isoelectric focusing and immunoassay tests. The detection is technically difficult due to the extent of similarity between the recombinant proteins and their endogenous counterparts. 

On the other hand the emergence of gene therapy as a result of recent advances in genetics science has raised concerns over the possibility of genetic enhancement of athletes, commonly referred to as “gene doping”. In response to this potential misuse of gene therapy, WADA declared that the non-therapeutic use of cells, genes, genetic elements or the modulation of gene expression having the capacity to enhance athletic performance, is prohibited. EPO, hGH, insulin-like growth factor-1 (IGF- 1), peroxisome proliferator-activated receptor-delta (PPAR delta) and myostatin inhibitor genes have been identified as primary targets for gene doping. 

Besides making the competitions unfair, gene doping puts the athletes health in great danger. Risks associated with gene doping fall into two main areas: 

First, the product and the procedures for delivery of the product are risky. The production of viral vectors requires considerable purification and testing for replication-competent viruses. Secondly, the uncontrolled expression of the genes may itself be harmful. 

At present, detection of doping in sport is based on two approaches. The main and currently most important approach is the detection of the specific substance itself and the second approach is the detection of the consequences of administering the doping agent. 

Recombinant proteins such as EPO and hGH can be detected by analysis of the isoforms of these hormones. However, genetic modifications will be very difficult to detect. For example, expression of antisense reagents within muscles to reduce the levels of myostatin will be hard to detect, as levels of circulating myostatin are low in man. Detection of transferred genetic materials may pose a problem, as current doping detection relies on urine and blood samples and it is generally considered that any form of tissue biopsy would be unacceptable. 

Since no actual abuse of gene doping has been documented so far, the prospects for gene doping remains essentially theoretical at present. However, this field as a whole should continue to be closely monitored. Despite the risks associated with untested gene-doping procedures and products, it seems quite possible that some athletes will be tempted to experience this type of doping. 

In order to pre-empt any possible exploitation of gene doping and recombinant drugs, extensive research is clearly required in these fields to ensure that competent and reliable methods are developed for the containment of these new trends of drug and therapeutic abuses in sport. 

*Dr. Farzad Kobarfard is currently working as an Associate Professor of Medicinal Chemistry of the Department of Medicinal Chemistry, School of Pharmacy, Shaheed Beheshti University of Medicinal Sciences, Tehran, Iran. He could be reached at the following e-mail address: farzadkobarfard@sbmu.ac.ir*

